# Upper Limb Paralysis as Presentation of Hepatocellular Carcinoma Bone Metastases

**DOI:** 10.7759/cureus.104556

**Published:** 2026-03-02

**Authors:** Patrícia Araújo, Mariana Cruz, Nuno Pardal, Carmélia Rodrigues, António Ferreira

**Affiliations:** 1 Internal Medicine, Unidade Local de Saúde do Alto Minho, Viana do Castelo, PRT; 2 Anatomical Pathology, Unidade Local de Saúde do Alto Minho, Viana do Castelo, PRT

**Keywords:** hepatocellular carcinoma (hcc), liver cirrhosis, neoplasm metastasis, neurologic deficits, spinal bone metastases

## Abstract

Hepatocellular carcinoma (HCC) is the most common primary liver malignancy and predominantly affects patients with cirrhosis. Extrahepatic metastasis is unusual, and when it occurs, it most often involves the lungs. Bone metastases are rare and associated with significant morbidity. We report the case of a 73-year-old man with alcoholic liver cirrhosis and HCC, initially presenting with two hepatic nodules. He underwent curative-intent surgical resection the following year. Two years later, he presented to the emergency department with several weeks of neck and bilateral shoulder pain, accompanied by a three-day history of right upper limb weakness. Imaging revealed multiple lytic vertebral lesions, including a pathological fracture at C6 with spinal cord compression, as well as additional thoracic and lumbar involvement and newly detected hepatic nodules. The patient underwent cervical radiotherapy, resulting in improved pain control. While the liver biopsy was inconclusive, the biopsy of a lumbar paravertebral mass confirmed metastatic HCC. Bone metastases from HCC may present as hypervascular soft tissue masses with cortical bone destruction, most commonly affecting the axial skeleton. This case highlights the importance of maintaining a high index of suspicion for metastatic disease in patients with prior HCC who present with persistent bone pain or neurological symptoms, even though HCC metastases are infrequent. Since the HCC incidence of recurrence is very high, close surveillance and prompt diagnostic evaluation are essential for early detection and timely intervention.

## Introduction

Hepatocellular carcinoma (HCC) is the most common primary liver cancer, accounting for 85-90% of cases globally [1]. Currently, it is the sixth most common cancer and the third leading cause of cancer-related death worldwide [2].

The majority of the cases, 80-90%, arise in the setting of cirrhosis due to chronic liver disease [3]. Therefore, the main risk factors for HCC include chronic hepatitis B virus (HBV) infection, hepatitis C virus (HCV) infection, and alcohol-related liver disease (ALD). It is important to note that HCC may also develop in non-cirrhotic livers, particularly in patients with chronic HBV infection or metabolic dysfunction-associated steatotic liver disease (MASLD) [1,3].

HCC often remains asymptomatic in its early stages, particularly when confined to the liver. Clinical manifestations usually emerge as the disease progresses and are frequently related to underlying cirrhosis rather than the tumor itself [3,4]. In more advanced stages, HCC may metastasize to distant organs, producing symptoms specific to the site of involvement. Risk factors for extrahepatic spread include elevated alpha-fetoprotein (AFP) levels (>400 μg/mL), vascular invasion, tumor size greater than 5 cm, and multifocal or infiltrative disease [5]. Extrahepatic metastases have been reported to be approximately 15%, the lungs being the most common site, in approximately one third of the cases [6]. Other locations involved are the peritoneum, adrenal glands, regional lymph nodes, and bone [3,5,6].

Although bone metastases are described in advanced HCC, presentation with a neurological deficit secondary to spinal cord compression is uncommon. We report a case of recurrent HCC presenting with upper limb paralysis due to vertebral metastasis with extensive soft tissue involvement, highlighting the diagnostic and prognostic challenges of this rare manifestation.

## Case presentation

A 73-year-old man presented to the emergency department with several weeks of neck and bilateral shoulder pain, progressive functional decline, and a three-day history of right upper limb weakness in November 2025.

**Figure 1:**
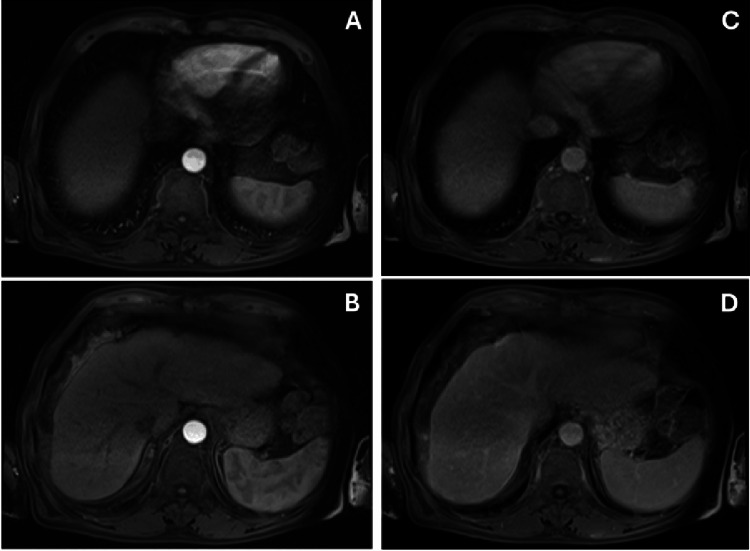
T1-weighted abdominal MRI images, performed six months after excision of hepatic nodules, with no visible focal lesions, showing no recurrence of hepatocellular carcinoma. A, B: arterial phase; C, D: venous phase

He had a medical history of stage 4 chronic kidney disease, alcoholic liver cirrhosis dating back to 2016, and HCC diagnosed in December 2022, with two hepatic nodules measuring 1.7 cm (segment IVa) and 5.5 cm (segment V). At the time of diagnosis, the largest lesion measured 5.5 cm, which formally exceeded the Milan criteria for liver transplantation. However, the patient had preserved liver function (Child-Pugh A) and no evidence of macrovascular invasion or clinically significant portal hypertension. Therefore, he was considered a candidate for surgical resection with curative intent. The patient underwent surgical resection of the two hepatic lesions in July 2023, without complications. After surgery, the patient was enrolled in regular surveillance with cross-sectional imaging every six months. Follow-up liver magnetic resonance imaging (MRI) in January 2024 showed no evidence of recurrence (Figure 1). However, between January 2024 and October 2025, surveillance was irregular due to poor outpatient follow-up adherence.

**Figure 2:**
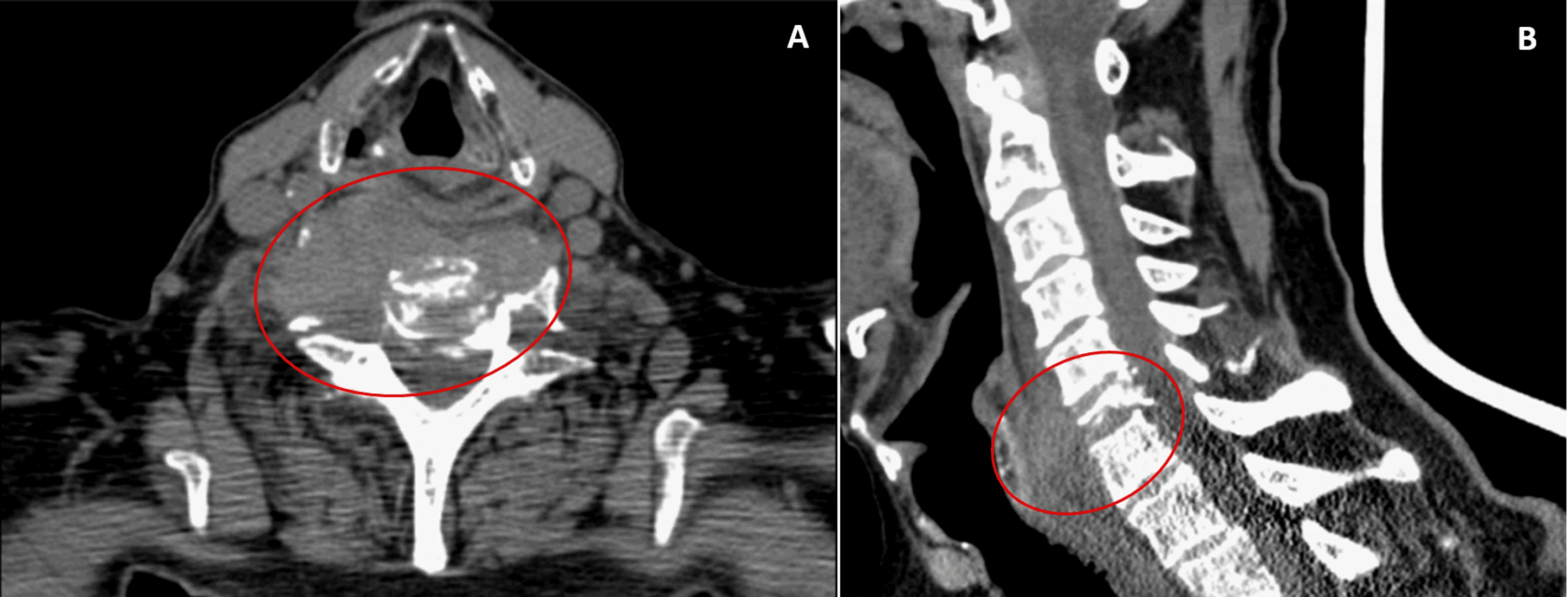
Cervical spine CT scan cervical showing the presence of an infiltrative soft tissue mass in the prevertebral topography of C6, and diffuse hypodensity of the body and right transverse process of the C6 vertebra with extensive lytic areas and cortical lysis (A); subtotal collapse of the vertebral body associated with pathological fracture, with marked reduction in the amplitude of the spinal canal at the C6 level with probable spinal cord compression (B).

**Figure 3:**
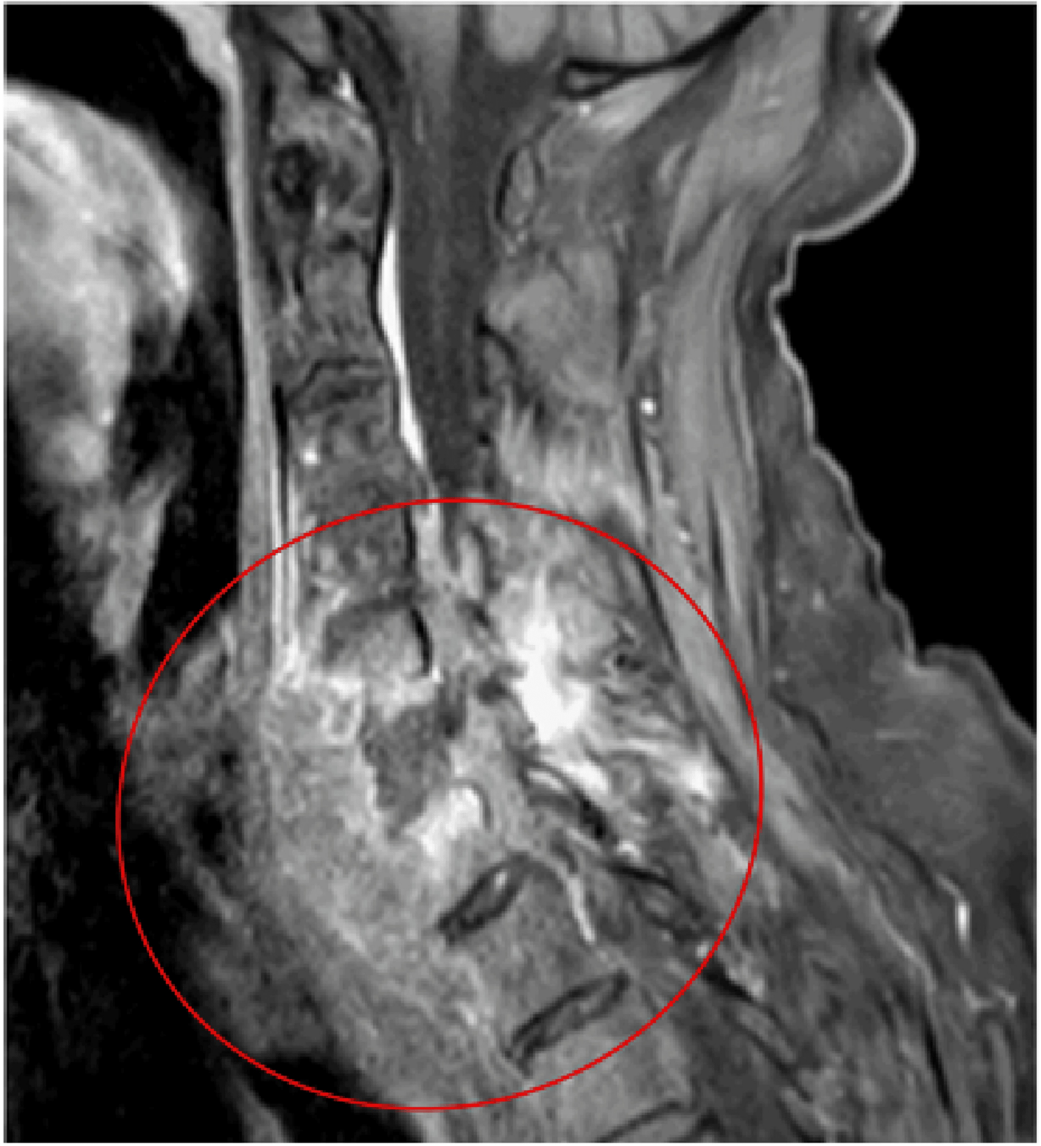
MRI of the cervical spine confirming severe spinal cord compression due to the lesion presente in the C6 vertebra measuring approximately 6.6x2.6 cm and extending from C5 to T1, causing compressive myelopathy.

In the current presentation, in November 2025, neurological examination revealed grade 2/5 muscle strength in the right forearm and grade 4/5 in the proximal right arm muscles. Deep tendon reflexes were brisk in the right upper limb, with preserved sensation to light touch and pinprick. No sphincter dysfunction was observed. Findings were suggestive of cervical spinal cord compression. Cervical spine computed tomography (CT) and MRI demonstrated a lytic lesion of the C6 vertebra with pathological fracture and severe spinal cord compression, associated with an infiltrative prevertebral soft tissue mass, measuring approximately 6.6x2.6cm, extending from C5 to T1 (Figures 2, 3).

Given the timing of symptom onset, the patient was managed conservatively following evaluation by Orthopedics and Neurosurgery. Dexamethasone 4mg every eight hours was initiated, and a cervical collar was applied. Laboratory evaluation revealed normocytic, normochromic anemia (hemoglobin 9.9 g/dL), without iron or vitamin deficiencies, subtle cholestasis, and markedly elevated erythrocyte sedimentation rate, with normal calcium, lactate dehydrogenase, immunoglobulins, and prostate-specific antigen levels; negative serology for HIV, HCV, and HBV (Table 1).

**Table 1:** Laboratory evaluation HBs: hepatitis B surface antigen; HCV: hepatitis C virus; INR: international normalized ratio

Parameters	Patient Value	Normal Range
Hemoglobin (g/dL)	9.9	13.2 - 17.2
Mean corpuscular volume (fL)	92.6	80.1 – 96.1
Mean globular hemoglobin (pg)	30.6	26.7 – 30.7
Leukocytes (10⁹/L)	6.34	4.0 – 10.0
Platelets (10⁹/L)	228	150 – 400
Erythrocyte sedimentation rate (mm)	91	2 – 8
Lactato desidrogenase (U/L)	220	125 – 220
Urea (mg/dL)	98	17 – 43
Creatinine (mg/dL)	2.33	0.8 – 1.3
Total bilirubin (mg/dL)	0.40	0.3 – 1.2
Alkaline phosphatase (UI/L)	199	30 – 120
Gamma glutamyl transferase (UI/L)	223	< 55
Aspartate aminotransferase (UI/L)	34	8 – 35
Alanine Aminotransferase (UI/L)	26	10 - 45
C-reactive protein (mg/dL)	<0.8	<0.51
Total proteins (g/dL)	6.9	6.4 – 8.2
HIV type 1-2	Non-reactive	Non-reactive
HBs antigen	Non-reactive	Non-reactive
Total HCV	Non-reactive	Non-reactive
Immunoglobulin A (mg/dL)	402	60 - 400
Immunoglobulin G (mg/dL)	1234	700 – 1600
Immunoglobulin M (mg/dL)	41	40 – 230
Protein electrophoresis	No abnormalities	-
Total calcium (mg/dL)	8.9	8.8 – 10.8
Prostate-Specific Antigen (ng/mL)	1.4	0 – 4.0
INR	1.31	-
Alpha-fetoprotein (ng/mL)	19106.8 (N <13.4)	< 13.4

**Figure 4:**
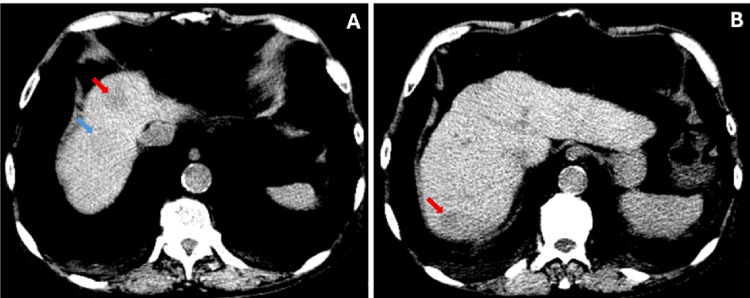
Abdominal CT scan without contrast in November 2025 revealed multiple hypodense hepatic nodules, with the largest ones in (A) segments IVa and VIII (red arrow and blue arrow, respectively) and in (B) segment VII, which were not present in the MRI performed in January 2024.

Review of prior records identified an abdominal ultrasound performed one month earlier that described a new hepatic nodule measuring approximately 2 cm. Subsequent abdominal-pelvic CT revealed multiple hepatic nodules, the largest measuring 28 mm in segment VII (Figure 4).

**Figure 5:**
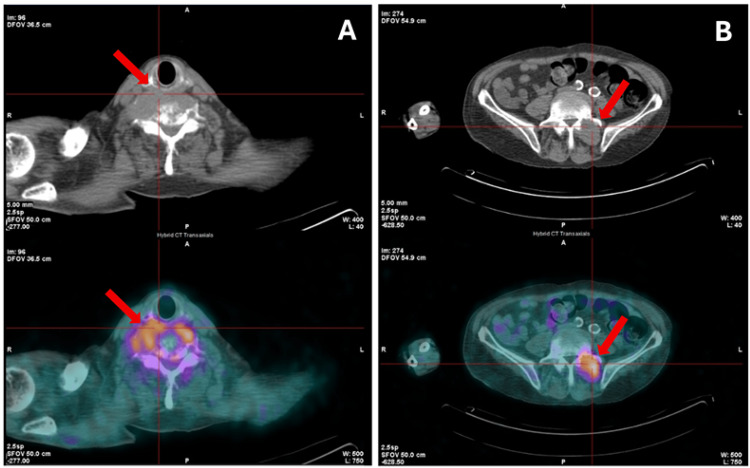
PET scan images revealing hypercaptation suggestive of a high-metabolic neoplastic lesion at C6 (A) and L4 (B) levels.

The patient was hospitalized in the Internal Medicine ward for further evaluation of liver nodules and bone metastases. A biopsy of a hepatic lesion was inconclusive, although with suspicion of neoplastic cells, and a biopsy of the cervical soft tissue mass revealed normal tissue (fibrous, muscular, and nervous), not representative of the lesion. Due to severe pain and spinal cord compression, the patient underwent five sessions of palliative cervical radiotherapy (five fractions of 5 Gy each, resulting in a total dose of 25 Gy), achieving partial pain relief. AFP was approximately 19,000 ng/mL. A positron emission tomography (PET) scan demonstrated high-metabolic lesions at C6, T2, L3-L5, with a soft tissue component at C6 and L4-L5, supporting the presence of active metastatic disease (Figure 5).

**Figure 6:**
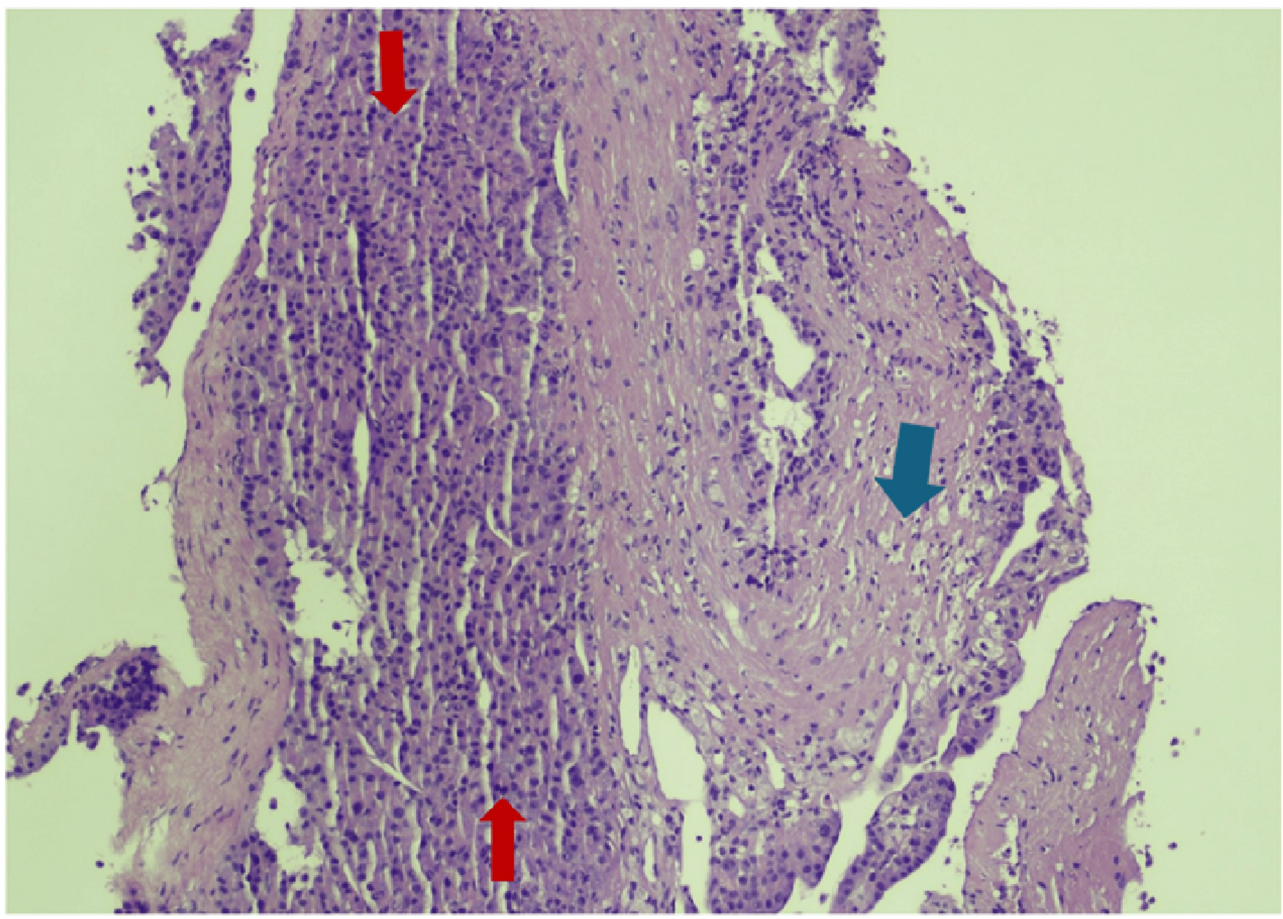
Histopathological evaluation of a left paravertebral soft tissue mass biopsy revealing infiltration of fibrous tissue (marked with a blue arrow) by hepatocellular carcinoma metastasis (indicated by a red arrow) (H&E, 100x).

**Figure 7:**
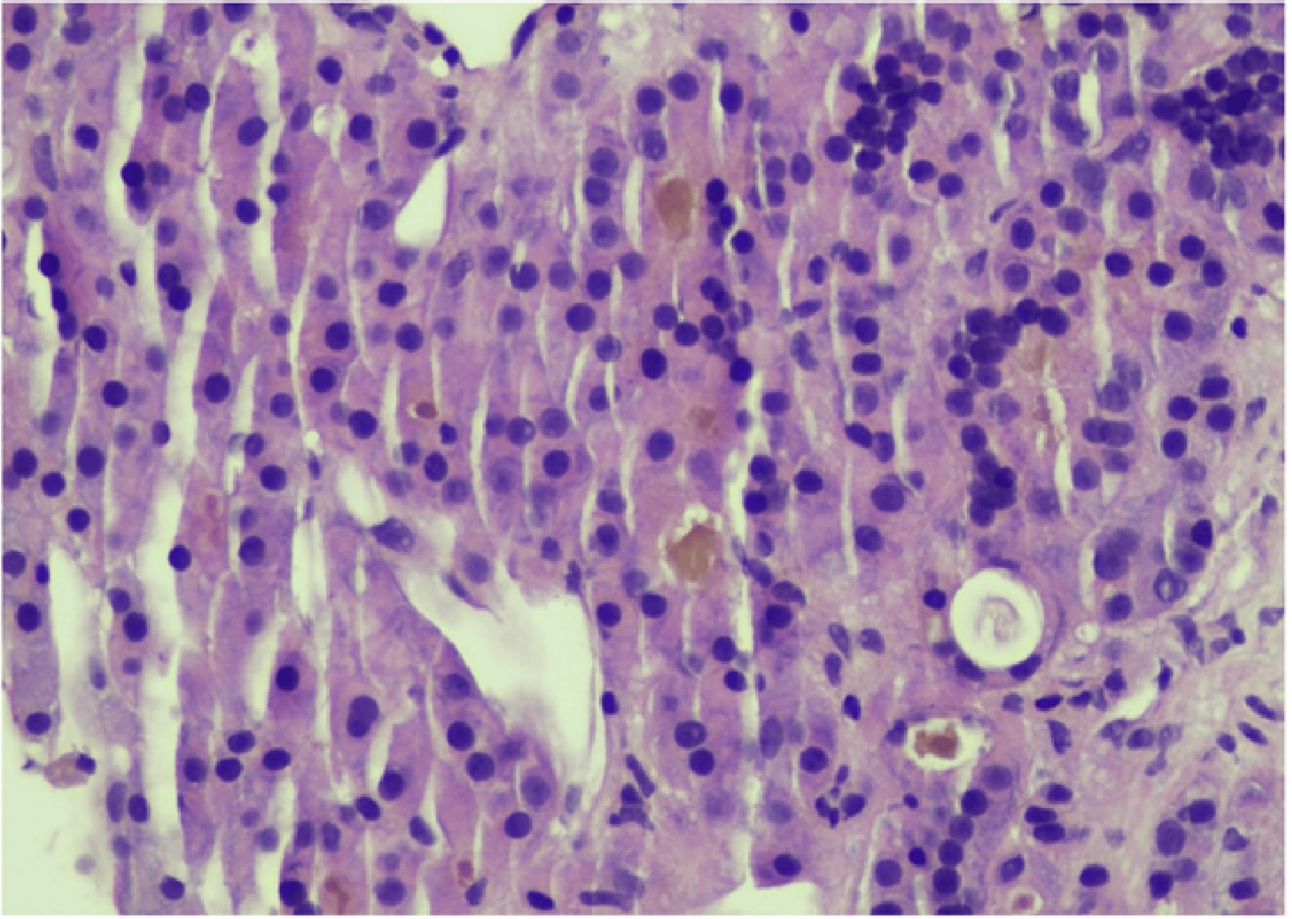
Hepatocellular carcinoma metastasis at higher power demonstrating trabecular and pseudoacinar morphology. The dark brown/yellow pigmented material is bile (H&E, 400x).

Biopsy of a left paravertebral soft tissue mass at the L4 level confirmed infiltration by HCC (Figures 6, 7) and excluded alternative primary malignancies.

Recurrence of HCC with bone metastases associated with soft tissue components was assumed. Despite optimized analgesia, neurological deficits persisted, and the patient experienced progressive functional decline.

Due to rapid neurological deterioration, poor functional status (Eastern Cooperative Oncology Group (ECOG) performance status 3-4), advanced metastatic burden, and underlying chronic kidney disease, the patient was deemed not a candidate for systemic therapy after multidisciplinary oncologic evaluation. He was discharged with referral to a home palliative care team and died in December 2025.

## Discussion

Bone metastases in HCC are relatively uncommon, with the reported prevalence ranging from 3% to 20% [7]. Most patients with bone metastases present with concomitant extraosseous metastatic disease; however, isolated or predominant skeletal involvement has been described [8].

Approximately 40% of bone metastases from HCC exhibit a hypervascular soft tissue component associated with cortical bone destruction and invasion of adjacent muscles or fat [9,10], as observed in this case. Extension to the soft tissue is much less frequently found in other metastatic bone diseases from less aggressive cancer types, such as breast or prostate cancer [11]. The axial skeleton, particularly the thoracic and lumbar spine, is the most frequently affected site, followed by the pelvic bone and ribs [9]. The majority of the patients with bone metastases from HCC suffer from skeletal-related events (SRE) as pathologic fractures and spinal cord compression. SREs often lead to severe pain, neurological symptoms, and marked deterioration in quality of life [8,9]. Management may include surgery, radiation therapy, and pain control, depending on disease extent and patient performance status [8,10].

HCC is a malignancy with a five-year survival rate below 20% and recurrence rates reaching up to 88% even after curative-intent treatment [12]. Although bone metastases after curative resection are uncommon (nearly 11.7%), their occurrence signals an aggressive disease course and poor prognosis, as seen in this case report, with a reported median survival of approximately 4.6 months [8,13].

In this case, serum AFP levels increased markedly to approximately 19,000 ng/mL, strongly supporting tumor recurrence despite initially inconclusive liver biopsy findings, a well-recognized limitation in metastatic HCC attributable to sampling error and intratumoral heterogeneity [14]. AFP levels above 400 ng/mL have been associated with higher tumor burden, vascular invasion, and increased risk of extrahepatic spread [15]. Although AFP alone is not diagnostic, when combined with compatible imaging findings and prior history of HCC, it significantly increases the suspicion of recurrent metastatic disease [16]. Furthermore, rapidly rising AFP levels during follow-up after curative-intent resection should prompt immediate investigation for recurrence, even in the absence of early imaging confirmation [17]. Extremely elevated AFP values, as observed in our patient, also correlate with aggressive tumor biology and poor prognosis [18].

The patient’s underlying alcoholic liver cirrhosis likely contributed to both tumor development and recurrence. Cirrhosis creates a pro-oncogenic microenvironment characterized by chronic inflammation, regenerative nodules, and genomic instability [19]. Moreover, cirrhotic patients often have limited hepatic reserve, which restricts therapeutic options at recurrence [16] and may partially explain the inability to pursue systemic therapy in this case. Alcohol-related liver disease has also been associated with more advanced-stage presentation and poorer overall survival compared to some viral etiologies [19].

Prognostic factors associated with poorer survival in HCC patients with bone metastases include high AFP levels, multiple skeletal lesions, poor performance status, and presence of extraosseous soft tissue components [11], all of which were present in our patient.

Similar cases of HCC presenting with bone metastases have been described, although they remain uncommon. Ruiz-Morales et al. reported two cases in which bone metastases were the initial manifestation of HCC [10], whereas in our case, metastases developed after an apparent curative resection. Compared with previously published cases, our patient demonstrated particularly aggressive disease, characterized by extensive axial skeletal involvement, prominent soft tissue components, and rapid neurological deterioration. Unlike isolated skeletal metastasis described in some reports, this case involved multifocal vertebral disease with spinal cord compression, emphasizing the need for urgent recognition of neurological red flags.

This case highlights an aggressive pattern of HCC recurrence presenting with extensive bone involvement, prominent soft tissue masses, and severe neurological compromise. Given the relative rarity of bone metastases in HCC, the diagnosis may be challenging, and multiple biopsies may be needed as histological confirmation is essential to exclude concomitant or alternative primary malignancies, even in the presence of markedly elevated AFP levels suggestive of invasive HCC.

## Conclusions

Although uncommon, skeletal metastases from HCC carry significant morbidity and poor prognosis. Clinicians should maintain a high index of suspicion in patients with prior HCC who present with new-onset bone pain or neurological symptoms, even after treatment with curative intent, given the high recurrence rate of HCC. Early recognition and histological confirmation are essential to guide appropriate palliative management and optimize quality of life.
